# Effects of Pulmonary Rehabilitation on Dyspnea, Quality of Life and Cognitive Function in COPD: A Systematic Review

**DOI:** 10.3390/jcm15020670

**Published:** 2026-01-14

**Authors:** Alessandro Vatrella, Angelantonio Maglio, Maria Pia Di Palo, Elisa Anna Contursi, Angelo Francesco Buscetto, Noemi Cafà, Marina Garofano, Rosaria Del Sorbo, Placido Bramanti, Colomba Pessolano, Andrea Marino, Mariaconsiglia Calabrese, Alessia Bramanti

**Affiliations:** Department of Medicine, Surgery and Dentistry, University of Salerno, Via S. Allende, 84081 Baronissi, Italyamaglio@unisa.it (A.M.); mariapia140497@gmail.com (M.P.D.P.); elisaannaspid@gmail.com (E.A.C.);

**Keywords:** pulmonary rehabilitation, Chronic Obstructive Pulmonary Disease, cognitive function, dyspnea, quality of life, respiratory muscle training, dysphonia, speech-language therapy, exercise training, rehabilitation outcomes

## Abstract

**Background/Objectives:** Chronic Obstructive Pulmonary Disease (COPD) is frequently associated with dyspnea, impaired health-related quality of life (HRQoL), and cognitive dysfunction. Although pulmonary rehabilitation (PR) is considered a core therapeutic strategy, its specific effects on cognitive function, dyspnea, and dysphonia remain unclear. This systematic review aimed to evaluate the impact of PR and respiratory or cognitive-focused rehabilitative interventions on dyspnea, quality of life, cognitive performance, and voice outcomes in adults with COPD. **Methods:** This review was conducted in accordance with PRISMA 2020 guidelines and registered in PROSPERO (CRD420251131325). A systematic search of PubMed, Scopus and Web of Science identified studies published between 2010 and 21 August 2025. Eligible designs included randomized and non-randomized controlled studies, cohort, and mixed-method studies involving adults with COPD undergoing rehabilitative interventions targeting dyspnea, cognition, dysphonia, or swallowing. Outcomes included cognitive measures, dyspnea scales, voice parameters, and HRQoL indices. **Results:** Twelve studies (*n* ≈ 810 participants) met inclusion criteria. Most PR and exercise-based programs showed improvements in global cognition and executive functions, particularly when combined with cognitive training or high-intensity exercise modalities. Dyspnea improved consistently following short- to medium-term PR or respiratory muscle training, whereas low-frequency long-term programs yielded limited benefit. HRQoL improved across structured PR programs, especially in multidimensional interventions. Only one study assessed dysphonia, reporting transient improvements in maximum phonation time following inspiratory muscle training. No included study evaluated dysphagia-related outcomes. **Conclusions:** PR and respiratory muscle training can enhance cognition, dyspnea, and HRQoL in COPD, although evidence for dysphonia remains scarce and dysphagia is entirely unaddressed. Future high-quality trials should adopt standardized outcome measures, include long-term follow-up, and integrate voice and swallowing assessments within PR pathways.

## 1. Introduction

Chronic Obstructive Pulmonary Disease (COPD) is a heterogeneous respiratory disorder characterized by persistent symptoms such as dyspnea, cough, and sputum production resulting from structural alterations of the airways, alveoli, or pulmonary vasculature that lead to chronic, progressive airflow limitation [1]. It ranks among the leading causes of morbidity and mortality worldwide and significantly reduces patients’ quality of life. Beyond respiratory manifestations, COPD is frequently associated with cognitive impairment—particularly in memory and attention—which correlates with worse functional outcomes, longer hospital stays, more frequent exacerbations, and lower health-related quality of life [2]. Oropharyngeal dysphagia represents another underrecognized yet clinically relevant condition in COPD, arising from impaired breathing–swallowing coordination, reduced laryngopharyngeal sensitivity, and generalized muscle weakness that compromise swallowing safety and efficiency, predisposing to aspiration, respiratory exacerbations, and malnutrition, with cachexia affecting up to one quarter of patients [3,4]. Phonatory function is also frequently impaired: dysphonia has been described as a common condition associated with altered breathing patterns and a predominance of thoracic rather than diaphragmatic breathing. In a recent study, patients exhibited reduced vocal intensity and pitch, along with greater acoustic signal distortion compared with healthy controls. Diaphragmatic breathing depth correlated positively with acoustic parameters such as jitter and shimmer, indicating that respiratory mechanics directly influence vocal quality [5]. Overall, these findings suggest that in COPD, respiratory dysfunction interferes with both swallowing and phonation, revealing a complex involvement of the upper aerodigestive tract. Dyspnea remains one of the hallmark and most disabling symptoms of COPD, reflecting mechanical, neuromuscular, and perceptual factors related to pulmonary hyperinflation, reduced inspiratory capacity, and impaired ventilatory control. It has been identified as a multidimensional marker associated with peripheral muscle dysfunction, reduced exercise capacity, and decreased quality of life [6]. In this context, pulmonary rehabilitation (PR) stands as a cornerstone of COPD management. In this context, pulmonary rehabilitation (PR) represents a cornerstone of COPD management. PR combines exercise training, education, and nutritional and psychological support to enhance functional capacity, reduce dyspnea, and promote well-being; randomized controlled trials have demonstrated significant improvements in exercise tolerance, quality of life, and respiratory symptoms compared with usual care [7]. Moreover, structured exercise interventions within PR programs have been shown to improve attention, memory, and executive functions—especially in cognitively impaired patients at baseline—suggesting that exercise may also benefit cognitive performance, although protocol heterogeneity limits comparability [8]. Dysphagia management in COPD is primarily conducted by speech-language pathologists through multimodal, function-oriented approaches that integrate both clinical and instrumental assessments (such as the Volume-Viscosity Swallow Test, Videofluoroscopic Swallowing Study, and Fiberoptic Endoscopic Evaluation of Swallowing). Rehabilitation typically includes respiratory–swallow coordination training, Expiratory Muscle Strength Training (EMST), compensatory postures, bolus modification, and patient education. These interventions, often embedded within pulmonary rehabilitation programs, are designed to maintain swallowing safety, prevent respiratory complications, and can also contribute to improved voice quality by enhancing respiratory control. However, standardized clinical guidelines for the management of dysphagia in COPD are still lacking [9]. Altered respiratory mechanics and reduced subglottic pressure associated with COPD may also lead to dysphonia, characterized by decreased maximum phonation time and impaired vocal stability [10]. Collectively, these findings emphasize the importance of an integrated rehabilitative approach—including speech-language therapy—to restore pneumophonatory coordination, optimize expiratory efficiency, and improve both communication and quality of life. Given this multidimensional clinical framework, the present review deliberately adopts a broad, patient-centred perspective that encompasses dyspnea, cognitive function, dysphagia, and dysphonia as interrelated outcomes potentially influenced by pulmonary rehabilitation and allied rehabilitative interventions. Importantly, this scope was defined a priori based on clinical relevance rather than on the anticipated availability of published evidence. Despite growing interest, the available evidence on the rehabilitative management of dysphagia, dysphonia, and cognitive impairment in COPD remains fragmented and heterogeneous. This systematic review therefore aims to critically evaluate and synthesize the current evidence on the effectiveness of speech-language and rehabilitative interventions—including voice training, and cognitive or exercise-based programs—in adults with COPD, assessing their impact on vocal and cognitive outcomes, quality of life, and disease-related complications, and to identify knowledge gaps and guide future research in this emerging field.

## 2. Materials and Methods

### 2.1. Study Protocol

This systematic review was conducted in accordance with the PRISMA 2020 guidelines [11]. The review protocol was registered in the PROSPERO international prospective register of systematic reviews (ID: CRD420251131325, date: 21 August 2025). Articles were selected according to the literature available on the management of dysphonia, and cognitive impairment in adults with COPD through rehabilitative and speech-language interventions. Eligible study designs included randomized controlled trials (RCTs), quasi-randomized and non-randomized controlled studies, cohort and case–control studies, as well as mixed-method and qualitative studies. The PICO framework (Population, Intervention, Comparator, Outcome) was used to formulate the research question and guide the selection process (Table 1) [12]. All outcomes included in the PICO framework were defined a priori based on clinical relevance and multidisciplinary practice in COPD management. Importantly, outcomes were retained regardless of the subsequent availability of eligible studies, in order to avoid selective outcome reporting and to ensure transparent representation of current evidence gaps.

### 2.2. Search Strategy and Study Selection

The literature search was performed electronically across four databases: PubMed, Scopus, Web of Science (WoS). Three independent reviewers (MG, EAC, MC) screened studies published between 1 January 2010 and 21 August 2025, using predefined search strategies that combined keywords and MeSH terms related to COPD, dysphagia, dysphonia, cognitive rehabilitation, and speech-language therapy. A summary of the electronic search strategies is presented in Table 2.

All records were imported into Rayyan software (https://www.rayyan.ai/, 2024 version). Duplicates were automatically removed, and any remaining duplicates were eliminated manually. Titles and abstracts were independently screened by two reviewers (MG, EAC), while full texts of potentially eligible articles were assessed by the same reviewers. Discrepancies were resolved through discussion and consensus, with a third reviewer (AB) consulted when necessary. Eligible study designs included randomized controlled trials (RCTs), quasi-randomized and non-randomized controlled studies, cohort studies, case–control studies, cross-sectional, mixed-method, and qualitative studies. Inclusion criteria were as follows:

- Source: studies published in English between 1 January 2010 and 21 August 2025;
- Study design: RCTs, non-randomized controlled studies, cohort, case–control, cross-sectional, mixed-method, or qualitative designs;
- Population: adults (≥18 years) with a confirmed diagnosis of COPD, presenting with dysphagia, dysphonia, or cognitive impairment, regardless of disease severity;
- Intervention: any rehabilitative intervention targeting swallowing, voice, or cognitive function, including speech and language therapy, swallowing or voice therapy, cognitive rehabilitation, respiratory muscle training, pulmonary rehabilitation, exercise programs, and behavioral or compensatory strategies (e.g., postural adjustments, bolus modification, swallowing maneuvers);
- Outcomes: clinical and instrumental assessments of swallowing function (e.g., FEES, VFSS, V-VST, EAT-10), voice outcomes (e.g., Voice Handicap Index), cognitive performance (e.g., MMSE, MoCA), and secondary outcomes such as quality of life, adherence, healthcare utilization, or cost-effectiveness.

Exclusion criteria were as follows:

- Source: studies published before 2010, after 21 August 2025, or not available in English;
- Study design: case reports, case series, editorials, letters, narrative reviews, and expert opinions;
- Population: studies not including patients with a confirmed diagnosis of COPD, or including participants with primary neurological, oncological, or other disorders as the main cause of dysphagia, dysphonia, or cognitive impairment (e.g., stroke, head and neck cancer, dementia);
- Intervention: pharmacological or surgical interventions without a rehabilitative component;
- Outcomes: studies not reporting any functional, clinical, or quality-of-life outcomes related to swallowing, voice, or cognition.

### 2.3. Data Extraction and Collection

Eligible studies were selected through agreement between the reviewers. In cases of disagreement during abstract screening, consensus was reached through discussion, and when needed, a third reviewer (MDP) was consulted to make the final decision. Data extraction was conducted independently by two reviewers following a standardized strategy aligned with the objectives of this review. Extracted data were organized according to predefined categories: (a) author, year (b) study design; (c) sample characteristics (number of participants, mean age, percentage of males); (d) COPD severity (GOLD stage, FEV_1_, CAT or mMRC scores); (e) intervention; (f) comparator; and (g) Key findings (related to cognitive outcomes, respiratory function and other relevant clinical measures) (Table 3). This structured methodology ensured accuracy and consistency in data collection, enabling a clear and comprehensive synthesis of results across studies.

### 2.4. Quality Assessment

The risk of bias of all included studies was assessed independently by two reviewers (MG, AB). Any discrepancies were resolved through discussion with a third reviewer (MC) until agreement was achieved. For randomized clinical trials, the Cochrane Risk of Bias 2 (RoB 2) tool [24] was employed. This instrument evaluates five methodological domains: adequacy of the randomization process, deviations from the intended interventions, completeness of outcome data, accuracy of outcome measurement, and risk of selective reporting. Each domain was classified as “low risk”, “some concerns”, or “high risk of bias” (Table 4).

**Table 5:** ROBINS-I assessment for non-randomized studies Abbreviations: PY = Probably Yes; P = Possibly; PN = Probably No.

Article	Bias Due to Confounding	Bias in Selection of Participants	Bias in Classification of Interventions	Bias Due to Deviations from Intended Interventions	Bias Due to Missing Data	Bias in Measurement of Outcomes	Bias in Selection of Reported Results	Overall Risk of Bias
Andrianopoulos et al., 2021 [13]	PY	P	PN	P	P	P	P	SERIOUS
France et al., 2021 [16]	PY	P	PN	PN	P	P	PN	SERIOUS
Kotani et al., 2025 [17]	PY	PY	PN	P	P	P	P	SERIOUS
Park et al., 2021 [19]	P	P	PN	P	P	P	PN	MODERATE

## 3. Results

### 3.1. Study Selection

The study selection process followed the PRISMA 2020 guidelines (See Supplementary Materials for the PRISMA 2020 checklist and abstract checklist). A total of 726 records were identified through database searches: 233 from PubMed, 313 from Scopus, and 180 from Web of Science. After the removal of 219 duplicates, 507 records were screened by title and abstract.

Following title and abstract screening, 480 articles were excluded because they did not meet the inclusion criteria. The remaining 27 full-text articles were assessed for eligibility.

Of these, 12 full-text studies were excluded for the following reasons:

- No rehabilitative intervention targeting dysphagia, dysphonia, cognition, or dyspnea (n = 6);
- Electrical stimulation only without active rehabilitative treatment (n = 1);
- Ventilatory support only, no rehabilitation (n = 1);
- Study protocols without implemented interventions (n = 5);
- Year < 2015 (n = 2).

**Figure 1:**
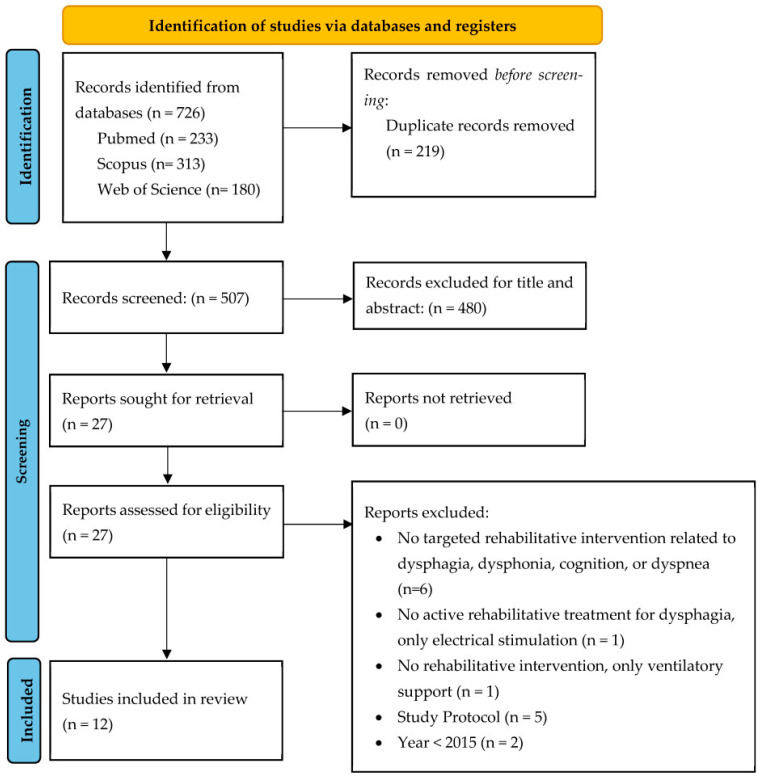
PRISMA flow chart.

Ultimately, 12 studies met the eligibility criteria and were included in the review. These studies specifically evaluated the effects of speech-language therapy, respiratory coordination training, inspiratory/expiratory muscle training, cognitive rehabilitation, or PR programs with speech-related outcomes in adults with COPD. However, none of the included studies reported dysphagia-specific outcomes, which stands in contrast to the initial review protocol that intended to analyse rehabilitative interventions targeting swallowing function. This gap highlights a lack of evidence on swallowing-focused rehabilitation in COPD populations. The complete selection process is illustrated in the PRISMA flow diagram (Figure 1).

### 3.2. Participants’ Demographics

The review encompassed 12 studies involving a total of approximately 810 patients, with mean ages generally ranging between 65 and 75 years. Sample sizes varied considerably, ranging from small pilot trials [10,14,20], to larger randomized or observational cohorts [16,18]. Most populations consisted of older adults, with a predominance of males [14,15,17]. From a clinical standpoint, the majority of patients were classified within GOLD stages II–III, with mean FEV_1_ values ranging from 36% to 55% of predicted [13,16,18,22]. Some cohorts also included individuals with more advanced disease (GOLD IV) [15,16], while the longitudinal study by Kotani et al. [17] documented progression from milder to more severe stages over a two-year period. Disease severity was primarily assessed using GOLD classification, FEV_1_, CAT or mMRC scores, whereas comorbidities and pharmacological treatments were inconsistently reported. Interventions predominantly involved PR, either alone or combined with adjunctive strategies such as cognitive training [22], inspiratory muscle training [15], high-intensity or combined exercise programs [14,20], cognitive-behavioral therapy [18], home-based cognitive rehabilitation [19], working memory training [23] or abdominal breathing techniques [21]. Comparator conditions included usual care, standard PR, sham training or pre–post study designs. The studies were conducted across Europe [16,22,23], Asia [17,19,21] and North America [18,20], suggesting a strong predominance of European and Asian research settings.

### 3.3. Cognitive Outcomes

Across the 11 included studies, cognitive outcomes were primarily assessed using validated tools such as the MoCA, MMSE, ACE-R, and neuropsychological domain-specific tests. Most interventions incorporating PR, either standalone or combined with cognitive or respiratory training, demonstrated modest yet clinically meaningful improvements in global cognition or specific cognitive domains. Short-term PR improved global cognition and domain-specific functions (memory, visuospatial, fluency) in both cognitively impaired and cognitively normal patients [13], while high-intensity aerobic plus resistance training enhanced executive functions, delayed recall, visuospatial ability and fluency beyond aerobic training alone [14]. Respiratory muscle training increased MMSE scores, although adding expiratory training did not produce additional benefits [15]. France et al. [16] reported no overall MoCA improvement following PR, yet patients with baseline cognitive impairment showed significant gains. Long-term, low-frequency outpatient PR preserved global and executive cognition despite physical decline [17], suggesting a stabilizing rather than enhancing effect. Pharmacological and behavioral interventions also showed cognitive benefits. Lavoie et al. [18] observed significant MoCA improvements across all treatment groups, particularly in individuals with higher physical activity levels. Home-based cognitive rehabilitation produced sustained cognitive improvements at 4-week follow-up [19]. Exercise modalities such as high-intensity interval training and continuous training improved visuospatial and attentional functions, with delayed MoCA gains only after one year [20]. Song et al. [21] demonstrated that head-down strong abdominal breathing combined with PR achieved greater MoCA improvements than control breathing strategies. A combined PR and cognitive training program yielded superior gains in MoCA and neurophysiological P300 markers compared to PR alone, suggesting synergistic effects [22]; in contrast, working memory training improved only task-specific abilities without transfer to global cognition [23]. Notably, among the cognitive assessment tools used, MoCA appeared more sensitive than MMSE in detecting changes in executive, attentional, and visuospatial domains, which are particularly relevant in COPD and non-amnestic cognitive impairment, although direct head-to-head comparisons between tools were limited. Overall, the evidence indicates that cognitive function in COPD can be improved or at least preserved through structured PR, especially when combined with cognitive or respiratory-focused interventions. The greatest cognitive improvements were observed in multidomain or integrative approaches, exercise and cognitive tasking, whereas isolated cognitive training or pharmacological interventions produced limited or task-specific effects (Table 6).

### 3.4. Dysphonia

Dysphonia was assessed only in one article included in this systematic review. Gracioli et al. [10] worked on two groups and administered a program of Low-intensity IMT. Training was performed at a load of 30% of the Maximal Inspiratory Pressure (MIP) for the IMT Group, and 10% of the MIP for the Simulated Inspiratory muscle training (SIMT) Group. Immediately after the treatment, there is a significant increase in subjects of the IMT group (10.2 before and 11.2 immediately after). As we can see from Table 7, MPTC is the only variable that differed significantly between the groups, so, we can affirm that the treatment show immediate positive results, but these are not confirmed 30 days after the intervention.

### 3.5. Dyspnea Outcomes

Across the included studies, dyspnea showed variable responses to pulmonary rehabilitation and respiratory muscle training interventions. In the randomized controlled trial by Cheng et al. [15], all groups undergoing respiratory muscle training (RMT) demonstrated a reduction in dyspnea. The combined inspiratory and expiratory muscle training group (IMT + EMT) showed the largest decrease in mMRC score (from 1.63 ± 0.98 to 1.13 ± 0.67; *p* < 0.01), while improvements in the IMT-only group were smaller and not statistically significant (*p* = 0.667). In the study by France et al. [16], dyspnea assessed using the CRQ-Dyspnea domain improved in both cohorts after six weeks. The AECOPD group, undergoing spontaneous recovery, showed an increase from 1.92 to 2.66, whereas the stable COPD group participating in PR improved from 2.58 to 3.36. The improvement was greater in the post-exacerbation group compared to stable COPD in rehabilitation. Similarly, Gracioli [10] observed a small immediate reduction in mMRC scores after low-intensity inspiratory muscle training (IMT and SIMT). However, this effect was transient, with mMRC values returning to or slightly exceeding baseline after 30 days (IMT: 1.4 → 1.3 → 1.6; SIMT: 1.5 → 1.4 → 1.5), with no statistically significant changes. Conversely, long-term data [17] demonstrated a progressive worsening of dyspnea after a two-year, low-frequency PR program. The proportion of patients with moderate dyspnea (mMRC 2) decreased (38.1% → 23.8%), while those with severe and very severe dyspnea increased (mMRC 3: 14.3% → 19.0%; mMRC 4: 0% → 14.3%). Finally, Tabka et al. [22] found that the addition of cognitive training to PR resulted in a more noticeable reduction in exercise-induced dyspnea. Borg scores at peak exercise decreased from 5.8 to 4.5 in the intervention group versus 5.7 to 4.5 in the control group, although changes were not statistically significant (*p* > 0.05). Resting dyspnea remained largely unchanged in both groups (Table 8).

### 3.6. Quality of Life Outcomes

Across the included studies, PR and respiratory training programs consistently demonstrated positive effects on health-related quality of life in patients with COPD, although the extent of improvement varied by intervention type, duration, and intensity. Short-term PR (3 weeks) significantly improved both the physical and mental components of quality of life, as shown by Andrianopoulos et al. [13], with SF-36 Physical and Mental scores increasing in both cognitively normal and cognitively impaired patients. Similarly, Cheng et al. [15] reported a significant reduction in CAT scores after 8 weeks of inspiratory or combined inspiratory/expiratory muscle training, indicating an improvement in symptom burden; however, adding expiratory training offered no additional benefit compared to IMT alone. France et al. [16] observed clinically relevant improvements in both CAT and all CRQ domains (Dyspnea, Emotion, Mastery, Fatigue) in patients recovering naturally from AECOPD and in those completing a structured 6-week PR program. Improvements were greater in the PR group, with a larger reduction in CAT scores and higher increases across CRQ domains, confirming that structured rehabilitation is more effective than spontaneous recovery in enhancing respiratory-related quality of life. Conversely, long-term, low-frequency outpatient PR [17] did not produce meaningful changes in quality of life, with CAT scores remaining virtually unchanged over two years, suggesting that infrequent rehabilitation may be insufficient to sustain QoL benefits. Finally, Lavoie et al. [18] demonstrated that combining self-management education with pharmacological therapy—especially when paired with exercise—led to significant improvements in PHQ-9 scores, reflecting enhanced psychological well-being and overall quality of life (Table 9).

## 4. Discussion

The findings of this systematic review suggest that PR and respiratory-focused interventions may positively influence cognitive performance, dyspnea, and HRQoL in patients with COPD, while evidence on dysphonia remains extremely limited. These results are encouraging but should be interpreted with caution considering methodological variability, sample size limitations, and risk of bias across the included studies. Furthermore, although dysphagia was included as an outcome of interest in the original review protocol and represents a clinically relevant issue in COPD, no eligible studies investigated swallowing function or dysphagia-specific rehabilitation. This absence of evidence should not be interpreted merely as a limitation of the present review, but rather as a critical clinical blind spot in current respiratory rehabilitation research. Despite the well-documented prevalence of oropharyngeal dysphagia in COPD and its association with aspiration risk, exacerbations, and nutritional decline, swallowing outcomes remain systematically neglected in interventional pulmonary rehabilitation trials, highlighting a significant gap in the literature. Finally, particularly in non-randomized studies in which the overall risk of bias was rated as serious, improvements in cognitive performance and health-related quality of life may be partially influenced by residual confounding factors such as structured supervision, increased social interaction, and behavioural engagement inherent to pulmonary rehabilitation settings. In contrast, improvements in dyspnea are more likely to reflect direct physiological effects of exercise and respiratory training. These considerations should therefore be taken into account when weighing the overall strength and interpretation of the reported findings.

### 4.1. Cognitive Outcomes

Cognitive outcomes were assessed in most of the included studies and showed generally favourable trends following PR. These findings are particularly relevant if considered in the context of the high prevalence of cognitive disorders in COPD. Recent evidence from a large meta-analysis [26] demonstrated that individuals with COPD have a 39% higher risk of developing cognitive impairment or dementia compared to healthy controls, especially the non-amnestic subtype of mild cognitive impairment (na-MCI), which affects executive functions, attention and visuospatial abilities rather than memory alone. These cognitive deficits are thought to be driven by chronic hypoxemia and impaired cerebral perfusion [7,27], systemic inflammation involving IL-6, TNF-α and CRP [28,29], and cardiovascular comorbidities frequently coexisting in COPD [30,31,32]. Physical inactivity and muscular deconditioning may further exacerbate neural vulnerability by reducing neuroplasticity and cerebral oxygen delivery [33,34]. However, the interpretation of cognitive outcomes is complicated by substantial heterogeneity in the nature of the interventions broadly labelled as “cognitive training.” In several studies, cognitive performance was assessed following structured pulmonary rehabilitation or exercise-based interventions without the inclusion of a dedicated cognitive-training component, suggesting that observed cognitive improvements were largely mediated indirectly through physical exercise, improved oxygenation, or behavioural engagement [14,16,20]. In contrast, only a limited number of studies implemented explicit cognitive rehabilitation protocols, such as structured programmes targeting attention, memory, language, visuospatial abilities, executive function, and problem solving delivered over multiple supervised sessions [22], or pulmonary rehabilitation combined with a defined cognitive-training component [19]. Where specified, these interventions involved therapist-led, domain-oriented cognitive exercises rather than computerized or self-guided tasks. Nevertheless, the content, intensity, progression criteria, and targeted cognitive domains were not consistently reported across studies, limiting reproducibility and reducing the immediate clinical applicability of the findings. Within the studies included in this review, Andrianopoulos et al. reported that a 3-week PR program improved global cognitive function in both cognitively normal and cognitively impaired participants. France et al. [16] found that patients recovering naturally from acute exacerbation did not show cognitive improvement, while those undergoing PR displayed significant gains in MoCA scores, suggesting that rehabilitation actively contributes to cognitive recovery rather than merely reflecting spontaneous resolution. Cheng et al. [15] indirectly supported this relationship: although cognition was not directly assessed, improvements in dyspnea, respiratory muscle strength and functional capacity may contribute to enhanced cognitive engagement and reduced cognitive fatigue in daily life. Although baseline disease severity (e.g., GOLD stage, FEV_1_) and cognitive status were reported in several studies, their role as potential modifiers of intervention effects could not be systematically explored due to heterogeneity in study design and the lack of subgroup-level analyses. Nevertheless, a recurring pattern emerged whereby cognitive benefits appeared more pronounced in patients with baseline cognitive impairment, as also observed by France et al. [16] and Andrianopoulos et al. [13]. This observation, while not formally tested, has relevant implications for clinical stratification and suggests that individuals with greater cognitive vulnerability may derive proportionally greater benefit from structured pulmonary rehabilitation programmes. Importantly, although several studies reported statistically significant improvements in cognitive screening scores (e.g., MoCA or MMSE), the clinical meaningfulness of these changes remains uncertain. Minimal clinically important differences for these instruments are not well established in the context of pulmonary rehabilitation in COPD, and small score changes may not necessarily translate into meaningful functional improvements in daily life. Therefore, cognitive outcomes should be interpreted as preliminary indicators of potential benefit rather than definitive evidence of clinically meaningful cognitive recovery. Altogether, these results support the hypothesis that PR may help counteract early cognitive decline in COPD, particularly by improving oxygen delivery, reducing physical inactivity and enhancing neural respiratory drive.

### 4.2. Dysphonia

Dysphonia was the least explored domain: only one included study investigated phonatory outcomes. Gracioli et al. [10] reported that low-intensity IMT produced a short-term improvement in Maximum Phonation Time, but this effect was not maintained after 30 days. This suggests a potential, yet transient, benefit of respiratory muscle conditioning on phonatory support, likely mediated by enhanced subglottic pressure control and expiratory flow regulation. The background literature [35,36,37,38] confirms that dysphonia is common in COPD and related to airflow limitation, hyperinflation, chronic cough and steroid use, but these studies lack rehabilitative interventions and were excluded from this review. The scarcity of interventional data highlights a missed opportunity to address voice and communication within PR frameworks, despite its relevance to patient–therapist interaction, social participation and QoL [10].

### 4.3. Dyspnea

Dyspnea improved in most short- and medium-term interventions. France et al. [16] documented greater increases in CRQ-Dyspnea and larger reductions in CAT score in the PR group compared with spontaneous recovery following AECOPD, highlighting the superiority of structured rehabilitation. Similarly, Cheng et al. [15] reported significant reductions in mMRC and CAT after both IMT and combined IMT + EMT protocols. In contrast, Kotani et al. [17] showed that a low-frequency PR programme (one supervised session per month over two years) did not improve CAT scores and was associated with a shift toward higher mMRC grades, suggesting disease progression. This finding raises important concerns regarding the clinical adequacy of low-frequency “maintenance” models of pulmonary rehabilitation. The observed worsening of dyspnea over time suggests that sub-therapeutic training doses may be insufficient to counteract disease progression and may provide a false sense of protection for both patients and clinicians. In this context, the concept that “some rehabilitation is better than none” cannot be assumed to hold true when intervention intensity, frequency, and progression are inadequate. These findings support a more critical stance toward low-intensity maintenance programs and reinforce the need for rehabilitation models that ensure sufficient training load, regular supervision, and physiological stimulus to achieve meaningful and sustained symptom control. Overall, these findings indicate that dyspnea relief depends on adequate training intensity, regular supervision, and sufficient respiratory load, rather than sporadic or low-dose interventions. This aligns with pathophysiological models describing dyspnea in COPD as the result of increased inspiratory neural drive and neuromechanical dissociation, a mismatch between ventilatory demand and the capacity of the respiratory system to respond [39]. PR likely attenuates this imbalance by reducing dynamic hyperinflation, improving inspiratory muscle function, and enhancing ventilatory efficiency [39] and also external evidence further supports these mechanisms. Ong et al. emphasized the multidimensional nature of dyspnea, distinguishing sensory intensity from emotional distress and reinforcing the concept that dyspnea arises from combined disturbances in neural respiratory drive, mechanical loading, and affective processing [40]. Maltais et al. [41] and subsequent mechanistic studies confirmed that PR improves dyspnea through reductions in dynamic hyperinflation, improved diaphragm efficiency, and enhanced peripheral muscle oxidative capacity. However, improvements are not universal, domain-specific dyspnea components (air hunger, effort, chest tightness) are inconsistently evaluated, and emotional/affective dyspnea (e.g., fear, anxiety) may not improve proportionally to sensory dyspnea [40,42,43]. Importantly, the impact of rehabilitation on dyspnea remains heterogeneous. While short-term and adequately dosed programs show benefits, long-term low-frequency interventions [17] or non-supervised protocols may fail to produce meaningful change. This reinforces the need for standardized dyspnea assessment and multidimensional evaluation, considering both sensory and affective domains, as recommended in contemporary frameworks [39,40].

### 4.4. QoL

QoL improved consistently in structured and adequately dosed PR programs. Andrianopoulos et al. [13] reported significant increases in both physical and mental components of the SF-36, regardless of baseline cognitive status, highlighting that even short-duration PR (3 weeks) can enhance patient-perceived functional capacity. Similarly, France et al. [16] found significant improvements across all domains of the CRQ, with greater gains in the stable COPD cohort undergoing PR compared with patients recovering naturally from AECOPD, who showed only partial recovery. Cheng et al. [15] observed clinically meaningful reductions in CAT scores following 8-week inspiratory muscle training, with or without expiratory training, suggesting that even targeted respiratory interventions improve patient-perceived health status. In contrast, Kotani et al. [17] demonstrated that a low-frequency PR model (one supervised session per month for two years) failed to produce improvements in CAT scores, suggesting that insufficient training intensity and poor continuity may limit QoL benefits. These findings are supported by external epidemiological data showing that QoL deterioration in COPD is progressive and strongly associated with symptom burden, exacerbation frequency and physical inactivity [44]. Longitudinal cohort data further indicate that lower baseline QoL, particularly high CAT or SGRQ scores, predicts higher mortality and hospitalization risk [45]. Additionally, Scientific Reports data [46] show that poorer QoL is correlated with systemic inflammation, reduced handgrip strength and cognitive frailty, reinforcing the concept that QoL in COPD is multidimensional and reflects not only respiratory symptoms but also comorbid physical and neuropsychological impairments. Altogether, these findings suggest that QoL improvements are dependent on rehabilitation programs being intensive, structured and progression-based. Conversely, low-frequency, non-progressive or poorly supervised interventions appear insufficient to modify health-related QoL. Nonetheless, heterogeneity of measurement tools (CRQ, CAT, SF-36, SGRQ), variability in program duration and the absence of long-term follow-up in most studies limit generalizability and indicate the need for standardized, multidimensional QoL assessment in future trials.

Methodological quality and risk of bias influence the interpretation of these findings. Among randomized controlled trials, none were judged at high risk of bias, although most were rated as having “some concerns,” mainly due to unclear randomization procedures and lack of protocol preregistration. Only one study [23] was assessed as low risk across all domains. In contrast, non-randomized studies showed greater susceptibility to bias: Andrianopoulos et al. [13], France et al. [16], and Kotani et al. [17] were rated at serious risk due to uncontrolled confounding, non-random participant selection, and limited adjustment for baseline differences. These limitations, together with small sample sizes and heterogeneity of interventions, underline the need for cautious interpretation and reduce the certainty of evidence, particularly for long-term outcomes and less-explored domains such as dysphonia.

In summary, structured PR and respiratory muscle training appear to positively affect cognition, dyspnea, and quality of life in COPD, particularly when delivered intensively and consistently. Evidence for dysphonia is extremely limited but suggests potential for improvement through respiratory-based interventions. Future research should prioritise high-quality randomized trials, standardised outcome measures, longer follow-up periods, and greater inclusion of voice-related and cognitive rehabilitation components within multidisciplinary PR models.

### 4.5. Integrative Mechanisms Linking Cognition, Dyspnea and Quality of Life

Improvements in cognition, dyspnea, and health-related quality of life observed following pulmonary rehabilitation should not be interpreted as independent outcomes, but rather as interconnected manifestations of shared physiological and neurobiological mechanisms. Pulmonary rehabilitation may enhance cerebral oxygen delivery through improved ventilatory efficiency and reduced dynamic hyperinflation, counteracting chronic hypoxemia and impaired cerebral perfusion, which have been implicated in cognitive decline in COPD [7,27]. In parallel, increased physical activity and reduced systemic inflammation—particularly involving IL-6, TNF-α and CRP—may support neuroplasticity, synaptic efficiency, and cognitive resilience [28,29,33,34].

Reductions in dyspnea and physical deconditioning may further facilitate greater behavioural engagement, autonomy, and psychosocial well-being, thereby indirectly contributing to improvements in both cognitive performance and quality of life [6,33]. This integrative framework may help explain why multidimensional pulmonary rehabilitation programmes, combining exercise training, respiratory optimisation and behavioural engagement, tend to yield broader and more consistent benefits across cognitive, respiratory and patient-reported outcomes than isolated or single-component interventions [7,8].

## 5. Conclusions and Future Directions

This systematic review shows that structured pulmonary rehabilitation and respiratory muscle training can improve dyspnea, health-related quality of life and, in most studies, cognitive performance in patients with COPD. These benefits emerge particularly in short- and medium-term programmes delivered with adequate intensity, supervision and progression, whereas low-frequency or poorly dosed rehabilitation programmes should be interpreted with caution, as they may fail to prevent symptom progression and should not be considered equivalent to structured, adequately intensive pulmonary rehabilitation. From a translational perspective, however, it should be acknowledged that high-intensity or combined cognitive–rehabilitation models often rely on resource-intensive approaches that may be difficult to implement in routine clinical practice, particularly in outpatient or resource-limited settings. Future rehabilitation strategies should therefore aim to balance clinical effectiveness with feasibility, scalability and real-world applicability. Cognitive outcomes—assessed in the majority of included studies—suggest that PR may enhance attention, executive functions and global cognition, likely through improved ventilatory efficiency and cerebral oxygenation; however, heterogeneity in study design and outcome measures requires cautious interpretation. Accordingly, greater confidence can be placed in findings derived from RCTs, whereas results from non-RCTs, particularly those rated as having moderate to serious risk of bias, should be interpreted with appropriate caution and considered primarily hypothesis-generating. Evidence on dysphonia remains scarce, with only one study evaluating vocal outcomes, although voice impairment is a clinically relevant symptom in COPD and deserves greater attention. From a future trial design perspective, research should prioritize the systematic inclusion of swallowing and voice-related outcomes within pulmonary rehabilitation programs, given the high clinical relevance of dysphagia and dysphonia and their current underrepresentation in interventional studies. The adoption of standardized and validated assessment tools is strongly recommended to improve comparability across studies, including instrumental swallowing assessments (e.g., FEES or VFSS), validated voice outcome measures, and harmonized cognitive screening tools complemented by functional outcome measures. Moreover, intervention protocols should clearly define intensity, frequency, duration and progression criteria, as poorly specified or low-dose programs appear insufficient to produce sustained benefits. Finally, longer-term follow-up is needed to assess durability of effects and to inform optimal maintenance strategies in real-world clinical settings. Future research should therefore prioritise well-designed, adequately powered trials with standardized protocols and long-term follow-up, alongside multidimensional assessment of dyspnea and quality of life, including sensory, emotional and functional domains. It will be essential to include patients with cognitive impairment to better understand feasibility and treatment responsiveness in this vulnerable subgroup and to incorporate structured speech and voice assessments to clarify the impact of respiratory and laryngeal training on dysphonia. Further investigation into neurophysiological mechanisms—such as cerebral oxygenation, neural respiratory drive and brain plasticity—could help explain cognitive improvements. Finally, technology-assisted rehabilitation, including tele-rehabilitation, virtual reality and biofeedback, represents a promising tool to increase accessibility, adherence and home-based continuity of care.

## 6. Limitations

This systematic review presents several limitations that should be considered when interpreting its findings. Although 12 studies were included, substantial heterogeneity existed in terms of intervention duration, intensity, outcome measures, and sample characteristics, This variability limited direct comparisons between studies and did not allow for the performance of a meta-analysis, as pooling of data across diverse methodologies and outcome metrics would not have been methodologically appropriate. Importantly, such heterogeneity also reflects the inherently multidimensional and context-dependent nature of pulmonary rehabilitation, which integrates physical, behavioural, educational, and psychosocial components that are closely interrelated and difficult to disentangle. In this regard, attempting to isolate single “active” components may not fully capture the therapeutic complexity of rehabilitation interventions and may compromise their ecological validity. Only a portion of the studies were randomized controlled trials, and even among these, the risk of bias was frequently rated as “some concerns,” mainly due to unclear randomization procedures, lack of protocol preregistration, or insufficient blinding. Non-randomized studies further increased the risk of confounding, particularly regarding comorbidities, disease severity, pharmacological treatment, or baseline cognitive performance. Follow-up periods were generally short, preventing conclusions about long-term sustainability of improvements in dyspnea, cognition, or quality of life. Sample sizes were often small, reducing statistical power and increasing susceptibility to type II error. Moreover, outcome assessment was not standardized across studies—dyspnea was measured with tools such as mMRC, Borg, VAS, or CRQ-Dyspnea, while cognition was assessed using different neuropsychological tests—complicating data synthesis. Dysphonia was evaluated in only one included study, limiting the generalizability of conclusions in this domain. Additionally, only three databases were screened (PubMed, Scopus, an Web of Science). This decision was based on a preliminary exploratory search, which showed that these databases sufficiently covered the relevant literature. Finally, most interventions were delivered in highly controlled settings, which may not reflect real-world adherence or feasibility, and publication bias cannot be fully excluded.

## Figures and Tables

**Table 1:** PICO Framework for the systematic review.

Element	Description
Population (P)	Adults (≥18 years) diagnosed with Chronic Obstructive Pulmonary Disease (COPD), irrespective of disease severity, presenting with one or more of the following conditions: dysphagia, dysphonia, or cognitive impairment.
Intervention (I)	Any rehabilitative intervention targeting dysphagia, dysphonia, or cognitive impairment, including: Speech and language therapy, swallowing therapy and exercises, voice therapy, cognitive rehabilitation, respiratory muscle training, pulmonary rehabilitation and exercise programs, behavioral or compensatory strategies (e.g., postural adjustments, bolus modification, swallowing maneuvers)
Comparator (C)	Usual care or standard medical management for COPD; no intervention or wait-list control; placebo or sham intervention; alternative rehabilitative interventions; or baseline/pre-intervention assessments in within-subject designs.
Outcomes (O)	Primary outcomes: Swallowing function (clinical/instrumental assessment) Voice outcomes Cognitive function Secondary outcomes: Quality of life Adherence Healthcare outcomes Cost-effectiveness

**Table 2:** Database search strategies.

Database	Search String	Filters and Limits
PubMed	#1 (“Pulmonary Disease, Chronic Obstructive”[Mesh] OR “Lung Diseases, Obstructive”[Mesh] OR COPD OR “pneumological diseases” OR “lung disease” OR “lung diseases” OR “respiratory disease” OR “respiratory diseases” OR “chronic obstructive pulmonary disease”) AND #2 (“rehabilitation” OR “exercise” OR coordination) AND #3 (“deglutition” OR “dysphonia” OR “voice disorder” OR “vocal dysfunction” OR “vocal fatigue” OR “dysphagia” OR “swallowing disorder” OR “impaired swallowing” OR “cognitive decline” OR “cognitive impairment” OR “swallowing dysfunction”)	Language: English Publication years: 2010–2025
Scopus	(COPD OR “pneumological diseases” OR “lung disease” OR “lung diseases” OR “respiratory disease” OR “respiratory diseases” OR “chronic obstructive pulmonary disease”) AND (“rehabilitation” OR “exercise” OR coordination) AND (“deglutition” OR “dysphonia” OR “voice disorder” OR “vocal dysfunction” OR “vocal fatigue” OR “dysphagia” OR “swallowing disorder” OR “impaired swallowing” OR “cognitive decline” OR “cognitive impairment” OR “swallowing dysfunction”)	Language: English Publication years: 2010–2025 Document type: Article
Web of Science (WoS)	(COPD OR “pneumological diseases” OR “lung disease” OR “lung diseases” OR “respiratory disease” OR “respiratory diseases” OR “chronic obstructive pulmonary disease”) AND (“rehabilitation” OR “exercise” OR coordination) AND (“deglutition” OR “dysphonia” OR “voice disorder” OR “vocal dysfunction” OR “vocal fatigue” OR “dysphagia” OR “swallowing disorder” OR “impaired swallowing” OR “cognitive decline” OR “cognitive impairment” OR “swallowing dysfunction”)	Language: English Publication years: 2010–2025 Document type: Article

**Table 3:** Characteristics of included studies investigating speech-language, cognitive, or respiratory-related rehabilitation in adults with Chronic Obstructive Pulmonary Disease (COPD).

Author (Year)	Design	N (Mean Age ± SD), Male	COPD Severity (GOLD/FEV_1_)	Intervention	Comparator	Key Findings
Andrianopoulos (2021) [13]	Pilot non-randomized	N = 60; 67.7 ± 8.4 years; ≈58% male	GOLD 2–4; FEV_1_ ≈ 46–47%	PR, 3 weeks, 4 sessions per week	Pre–post; cognitively impaired vs. cognitively normal	Both groups improved cognition and QoL; domain-specific gains in both.
Aquino (2016) [14]	Randomized controlled trial	N = 28; 68.3 ± 9.6 years; 100% male	GOLD 2 (FEV_1_ 68.4%)	High-intensity aerobic + resistance training, 2 sessions per day, 5 days per week, 4 weeks	Aerobic training only	Combined training improved executive functions and memory more than aerobic alone.
Cheng (2022) [15]	Randomized controlled trial	N = 48; 67.2 ± 7.3 years; 100% male	IMT + EMT group: GOLD IV, FEV_1_ ≈ 29.5%; IMT-only group: GOLD III, FEV_1_ ≈ 41.2%	IMT + EMT, 30 breaths twice per day, 5 days per week, 8 weeks	IMT only	MMSE, CAT, mMRC and diaphragm thickness improved in both groups; no additional cognitive or functional benefit from EMT
France (2021) [16]	Prospective observational	N = 70; AECOPD 67.8 ± 9.3 years; PR 68.7 ± 6.7 years; male NR	AECOPD: GOLD 3 (FEV_1_ 41.2%); PR: GOLD 2 (FEV_1_ 55%)	Outpatient PR, 6 weeks, 2 sessions per week (exercise + education)	6-week natural recovery without rehabilitation (AECOPD group)	PR improved anxiety, CAT, physical function; MoCA improved only in cognitively impaired at baseline
Gracioli (2025) [10]	Randomized, single-blind trial	N = 17; 61.5 ± 7.1 (IMT), 62.8 ± 6.9 (Sham); 22–37% male	IMT group: GOLD III, FEV_1_ ≈ 45.6%; Sham: GOLD II, FEV_1_ ≈ 73.9%	Low-intensity IMT at 30% MIP, 3 sets × 15 breaths	Sham inspiratory muscle training at 10% MIP	Immediate increase in MPT/e/in IMT group; VoiSS decreased immediately in sham group; no effects sustained at 30 days; no immediate airflow-limitation change; MPTC differed between groups over time.
Kotani (2025) [17]	Retrospective longitudinal	N = 21; 76.4 ± 7.3 years; 100% male	Baseline GOLD 1–3; FEV_1_ 61.2% → progressed to GOLD 2–4 over 2 years	Low-frequency outpatient PR once per month + education, 2 years	Baseline vs. post–2 years	Cognitive and frontal function preserved; physical decline despite stable cognition.
Lavoie (2019) [18]	Randomized, partially double-blind, placebo-controlled trial	N = 304; 64.8 ± 6.6 years; 66.1% male	GOLD 2–4; FEV_1_ 48.7 ± 13.2% predicted	SMBM + one of tiotropium, tiotropium/olodaterol, tiotropium/olodaterol + exercise training, or placebo; 12 weeks	SMBM + placebo	Anxiety, depression and MoCA scores significantly improved in all groups; better anxiety outcomes with increased daily physical activity, and cognitive gains with greater exercise capacity.
Park, (2021) [19]	Quasi-experimental (non-randomized controlled study)	N = 60; 70.3 ± 7.5 years; 81.7% male	GOLD 1–4	Home-based cognitive rehabilitation, 6 sessions over 2 weeks	Usual care	Significant improvements in MoCA, COPD self-management ability, and QoL in the intervention group; cognitive and QoL benefits maintained at 4-week follow-up
Rosenstein (2021) [20]	Randomized pilot	N = 36; 67.5 ± 9 years; 36% male	GOLD 2; FEV_1_ 59%	HIIT vs. CT, 3 times per week, 12 weeks + education	Between-group comparison	Both groups improved visuospatial and attention performance; MoCA improvement observed only at 1-year follow-up; no significant cognitive differences between HIIT and CT
Song (2025) [21]	Randomized controlled trial (3-arm)	N = 63; ~66–67 years; HDBT 5/16 male/female	GOLD 2; FEV_1_/FVC ≈ 62%	HDBT, 60 min, 3 times per week, 12 weeks combined with PR	Two controls: (1) Head-down tilt only (HDT); (2) Abdominal breathing only (BT), both with identical schedule	HDBT improved MoCA scores and obstacle-walking gait (stride length & speed) more than HDT and BT. All groups improved cognition post-intervention; no gait differences during normal walking.
Tabka (2023) [22]	Randomized controlled trial	N = 39; Intervention: 65.1 ± 7.0 years, 56% male; Control: 64.3 ± 6.8 years, 55% male	GOLD III; mean FEV_1_ ≈ 36–38% predicted	PR + 20 min cognitive training, 3 days per week, 12 weeks	PR only	Both groups improved MoCA and exercise capacity; combined PR + cognitive training resulted in greater improvement in cognitive function.
van Beers (2021) [23]	Randomized, double-blind, placebo-controlled	N = 64; 66.2 ± 7.2 years; 45% male	Moderate COPD (GOLD II–III); FEV_1_: 58.5% (intervention), 60.6% (placebo) predicted	WMT: 12 weeks, 2–3 sessions per week (30 total), followed by 12 weeks of maintenance, 1 session per week (12 total). Computerized adaptive visuospatial, digit span, letter tasks.	Sham WMT: identical schedule, but fixed low-level task difficulty (non-adaptive).	Improved performance only in trained WMT tasks. No significant improvement in global cognition (ACE-R, CANTAB), physical capacity (6MWD), lifestyle behaviours, stress susceptibility, or healthy lifestyle goal recall.

Abbreviations: 6MWT, Six-Minute Walk Test; ACE-R, Addenbrooke’s Cognitive Examination–Revised; AECOPD, Acute Exacerbation of Chronic Obstructive Pulmonary Disease; BT, Breathing Training; CAT, COPD Assessment Test; CBT, Cognitive-Behavioral Therapy; CANTAB, Cambridge Neuropsychological Test Automated Battery; COPD, Chronic Obstructive Pulmonary Disease; CT, Continuous Training; EMT, Expiratory Muscle Training; FEV_1_, Forced Expiratory Volume in One Second; FEV_1_/FVC, Ratio of Forced Expiratory Volume in One Second to Forced Vital Capacity; FVC, Forced Vital Capacity; GOLD, Global Initiative for Chronic Obstructive Lung Disease; HDBT, Head-Down Strong Abdominal Breathing Training; HDT, Head-Down Tilt; HIIT, High-Intensity Interval Training; IMT, Inspiratory Muscle Training; MIP, Maximal Inspiratory Pressure; MMSE, Mini-Mental State Examination; MoCA, Montreal Cognitive Assessment; MPT, Maximum Phonation Time; MPT/e, Maximum Phonation Time on vowel /e/; MPTC, Maximum Phonation Time during Counting; mMRC, Modified Medical Research Council Dyspnea Scale; NR, Not Reported; PR, Pulmonary Rehabilitation; QoL, Quality of Life; RCT, Randomized Controlled Trial; SD, Standard Deviation; SMBM, Self-Management Behaviour Modification; SWAL-QoL, Swallowing Quality of Life Questionnaire; VoiSS, Voice Symptom Scale; WMT, Working Memory Training.

**Table 4:** Cochrane Risk of Bias assessment of individual randomized controlled studies. Risk of bias/concern was graded as: 🟡 low risk of bias; 🟢 some concerns; 🔴 high risk of bias. Overall risk of bias reflects the highest risk across domains.

Article	Randomization Process	Deviations from Intended Interventions	Missing Outcome Data	Measurement of Outcomes	Selection of Reported Results	Overall Risk of Bias
Aquino et al., 2016 [14]	🟡	🟢	🟢	🟢	🟡	🟡
Cheng et al., 2022 [15]	🟡	🟢	🟢	🟢	🟡	🟡
Lavoie et al., 2019 [18]	🟢	🟡	🟡	🟢	🟡	🟡
Gracioli et al., 2025 [10]	🟡	🟢	🟢	🟢	🟡	🟡
Rosenstein et al., 2021 [20]	🟡	🟢	🟢	🟢	🟡	🟡
Song et al., 2025 [21]	🟡	🟢	🟢	🟢	🟡	🟡
Tabka et al., 2023 [22]	🟡	🟢	🟢	🟢	🟡	🟡
van Beers et al., 2021 [23]	🟢	🟢	🟢	🟢	🟡	🟢

For non-randomized studies, the ROBINS-I tool [25] was used to evaluate the risk of bias. This instrument examines seven domains: potential confounding factors, selection of participants, accuracy in classifying interventions, deviations from the intended intervention, completeness of outcome data, reliability of outcome measurements, and selective reporting of results. Based on these criteria, each study was rated as having a “low”, “moderate”, or “serious” risk of bias (Table 5).

**Table 6:** Summary of studies evaluating the effects of pulmonary rehabilitation, respiratory training, or cognitive interventions on cognitive outcomes in COPD patients.

Study (Author, Year, Design)	Outcome Measures	Quantitative Cognitive Results	Narrative Summary
Andrianopoulos et al., 2021 [13]	MoCA, S-MMSE, T-ICS, ACE-R	MoCA: CI 23.24 → 23.38; CN 27.31 → 27.38. S-MMSE: CI 27.10 → 27.73; CN 28.37 → 28.93. T-ICS: CI 32.75 → 33.91; CN 35.37 → 36.62. ACE-R: CI 82.02 → 86.42; CN 92.07 → 93.95.	Short-term PR improved global cognition in both groups, with small-to-moderate domain gains (memory, visuospatial, fluency, executive functions).
Aquino et al., 2016 [14]	Attention Test, Rey Recall (IR/DR), Raven, Verbal Fluency, Drawing Test	Attention: 61.71 → 64.86 (*p* < 0.01). Rey-DR: 8.29 → 9.64 (*p* < 0.05 vs. control). Raven: 27.71 → 29.43 (*p* < 0.05). Fluency: 35.71 → 40.71 (*p* < 0.01).	Combined high-intensity aerobic + resistance training was superior to aerobic-only training in improving memory, executive functions, and visuospatial abilities.
Cheng et al., 2022 [15]	MMSE	24.39 ± 2.50 → 26.00 ± 4.13 (Δ + 1.61, *p* = 0.002). No difference between IMT vs. IMT + EMT (*p* = 0.585).	Respiratory muscle training improved MMSE scores; adding expiratory training offered no additional cognitive benefit.
France et al., 2021 [16]	MoCA	AECOPD: 24.04 → 23.23 (Δ − 0.8 ± 3.2; *p* = 0.205). PR Group: 24.39 → 24.82 (Δ + 0.6 ± 2.8; NS). Subgroup with baseline CI: +1.6 ± 2.4 (*p* = 0.004).	PR did not improve MoCA overall, but significantly enhanced cognition in patients with baseline cognitive impairment.
Kotani et al., 2025 [17]	MMSE, MoCA-J, FAB	MoCA-J: 23.9 ± 4.0 → 24.3 ± 4.1 (*p* = 0.722). MMSE: 28.5 → 28.3 (*p* = 0.122). FAB: 14.9 → 14.8 (*p* = 0.791).	Over 2 years, low-frequency outpatient PR maintained stable global and executive cognitive function despite progressive physical decline.
Lavoie et al., 2019 [18]	MoCA	All groups improved: Tiotropium/Olodaterol + Exercise: 25.7 → 27.7. CG: 25.8 → 27.2.	Significant MoCA improvement in all treatment arms; greater cognitive improvements observed in those with higher physical activity.
Park et al., 2021 [19]	MVCI-scale	Intervention: 21.97 ± 1.30 → 23.20 ± 1.24 → 23.73 ± 1.14. CG: no significant change.	Home-based cognitive rehabilitation significantly improved cognition and maintained effects at 4-week follow-up.
Rosenstein et al., 2021 [20]	MoCA, WAIS Digit Span, Rey–Osterrieth Complex Figure	MoCA: 25.7 → 25.6 → 26.3. Digit Span: 14.8 → 15.9 → 16.4. Rey-O: 29.1 → 30.8 → 30.0.	Improvements in visuospatial and attention memory after 12 weeks; MoCA improved only at 1-year follow-up. No difference between HIIT and continuous training.
Song et al., 2025 [21]	MoCA	MoCA HDBT: 18 → 24 (*p* < 0.001). MoCA HDT: 19 → 23. MoCA Breathing only: 19 → 23. Post-intervention comparison: HDBT > both controls (*p* < 0.05).	All groups improved MoCA, but head-down strong abdominal breathing showed the greatest cognitive gains.
Tabka et al., 2023 [22]	MoCA, P300	MoCA: +3.87 vs. +1.62 (*p* < 0.001). P300: improved latency/amplitude more in intervention group.	Combining PR with cognitive training resulted in greater cognitive and neurophysiological improvements compared to PR alone.
van Beers et al., 2021 [23]	CANTAB: (MOT, PAL, SST, RTI, DMS, SWM) Working Memory Span (training tasks): Visuospatial WM task, Backward digit span, Letter span	CANTAB MOT (ms): IG: 896 → 903 → 860; CG:894 → 865 → 827 PAL (errors): IG: 20 → 14 → 17; CG: 19 → 16 → 14.5 SST (ms): IG: 244 → 258 → 249; CG: 240 → 236 → 223 RTI—5-choice movement time (ms): IG: 290 → 282 → 272; CG: 283 → 309 → 307 DMS—Error given error (%): IG: 0.0 → 0.0 → 0.0; CG: 0.0 → 0.0 → 0.0 SWM—Between errors: IG: 16 → 15 → 16; CG: 15 → 17 → 12 Working Memory Span (training tasks) IG: ↑ significant improvement (Session 1 → 30, *p* < 0.001) → stable (Session 30 → 42, *p* = 0.399) CG: No improvement → no change → no change	Working memory training improved only the trained tasks, with no changes in the control group. Most CANTAB outcomes showed no significant effects, except a small improvement in RTI at follow-up. Overall, cognitive gains were limited to near-transfer, with no global cognitive benefits.

ACE-R, Addenbrooke’s Cognitive Examination—Revised; AECOPD, Acute Exacerbation of Chronic Obstructive Pulmonary Disease; CANTAB, Cambridge Neuropsychological Test Automated Battery; CI, Cognitive Impairment; CN, Cognitively Normal; COPD, Chronic Obstructive Pulmonary Disease; DMS, Delayed Matching to Sample; DR, Delayed Recall; EMT, Expiratory Muscle Training; FAB, Frontal Assessment Battery; HDBT, Head-Down Breathing Training; HDT, Head-Down Training; IMT, Inspiratory Muscle Training; IR, Immediate Recall; MMSE, Mini-Mental State Examination; MoCA, Montreal Cognitive Assessment; MoCA-J, Montreal Cognitive Assessment—Japanese version; MOT, Motor Screening Task; MVCI, Mini-Mental State Examination–Based Vascular Cognitive Impairment Scale; PAL, Paired Associates Learning; P300, Event-Related Potential P300 wave; PR, Pulmonary Rehabilitation; RTI, Reaction Time Task; S-MMSE, Standardized Mini-Mental State Examination; SST, Stop Signal Task; SWM, Spatial Working Memory; T-ICS, Telephone Interview for Cognitive Status; WAIS, Wechsler Adult Intelligence Scale.

**Table 7:** Summary of studies on dysphonia-related outcomes in COPD patients.

Study	Intervention (I)	Dysphonia Outcomes—Measures	Dysphonia—Quantitative Findings	Dysphonia—Key Findings
Gracioli et al., 2025 [10]	Low-intensity IMT group: 30% MIP resistance SIMT: 10% MIP; 3 sets × 15 breaths, 30 s rest, 15 breaths/min; PowerBreathe^®^ device	Maximum Phonation Time (MPT/e) and MPTC (Maximum Phonation Time after Counting)	MPT/e IMT: 10.2 ± 2.2 → 11.2 ± 2.4 → 8.0 ± 3.0 (*p* = 0.434) SIMT: 16.0 ± 3.6 → 16.4 ± 4.7 → 16 ± 5 (*p* = 0.088) MPTC IMT: 12.8 ± 3.2 → 12.9 ± 3.0 → 12.0 ± 4.0 (*p* = 0.824) SIMT: 16.0 ± 3.6 → 16.4 ± 4.7 → 16.0 ± 5.0 (*p* = 0.003)	No significant differences between groups immediately post-intervention. IMT group showed a transient increase in MPT/e immediately after training, but not sustained at 30 days. SIMT group showed a significant change in MPTC over time (*p* = 0.003). No long-term improvements; authors suggest longer duration

Abbreviations: IMT, Inspiratory Muscle Training; SIMT, Sham Inspiratory Muscle Training; MIP, Maximal Inspiratory Pressure; MPT/e, Maximum Phonation Time on sustained vowel /e/; MPTC, Maximum Phonation Time after Counting; COPD, Chronic Obstructive Pulmonary Disease.

**Table 8:** Summary of studies evaluating dyspnea outcomes in COPD patients.

Study (Author, Year)	Dyspnea Outcome Measure	Quantitative Results	Narrative Summary
Cheng et al., 2022 [15]	mMRC score	mMRC before RMT treatment = 1.63, SD ± 0.98—after RMT treatment = 1.13, SD ± 0.67 (*p* < 0.01). mMRC Group 1 = 1.07 ± 0.72; mMRC Group 2 = 1.20 ± 0.62 (*p* = 0.667).	All training modalities reduced dyspnea; combined IMT + EMT yielded the best improvement.
France et al., 2021 [16]	CRQ-Dyspnea	Cohort 1 (AECOPD): Mean = 1.91979 → 2.66247 Cohort 2 (PR): Mean = 2.57964 → 3.35640	Dyspnea improved in both groups. Mean CRQ-Dyspnea increase was greater in the AECOPD group than in stable COPD receiving PR.
Gracioli et al., 2025 [10]	mMRC	IMT group: 1.4 → 1.3 → 1.6 (SD ±1.8/±1.7/±1.6), *p* = 0.708 SIMT group: 1.5 → 1.4 → 1.5 (SD ±1.4/±1.2/±1.4), *p* = 0.611	Both groups showed a slight improvement in dyspnea immediately after treatment, followed by a mild worsening at 30 days. Changes were not statistically significant.
Kotani et al., 2025 [17]	mMRC	mMRC after 2-year PR program: Decrease in mild dyspnea (Grade 1: 33.3% → 28.6%) Decrease in moderate dyspnea (Grade 2: 38.1% → 23.8%) Increase in more severe dyspnea: Grade 3: 14.3% → 19.0% Grade 4: 0% → 14.3%	Despite the 2-year PR program, dyspnea did not improve. The proportion of patients with mild-to-moderate dyspnea (Grades 1–2) decreased, while those with severe-to-very severe dyspnea (Grades 3–4) increased. Low-frequency PR (once monthly) may be insufficient to maintain or improve dyspnea.
Tabka et al., 2023 [22]	Borg scale	Intervention Group: Borg at Rest: before 1.8 ± 0.3—after 1.6 ± 0.5 (*p* = 0.9) Borg at Peak Exercise: before 5.8 ± 0.8—after 4.5 ± 0.5 (*p* = 1.0) Control Group: Borg at Rest: before 1.9 ± 0.5—after 1.7 ± 0.4 (*p* = 0.9) Borg at Peak Exercise: before 5.7 ± 0.9—after 4.5 ± 0.8 (*p* = 0.9)	CT added to PR led to greater improvement in exercise-induced dyspnea.

Abbreviations: mMRC, modified Medical Research Council Dyspnea Scale; RMT, Respiratory Muscle Training; IMT, Inspiratory Muscle Training; EMT, Expiratory Muscle Training; CRQ, Chronic Respiratory Questionnaire; AECOPD, Acute Exacerbation of Chronic Obstructive Pulmonary Disease; PR, Pulmonary Rehabilitation; SIMT, Sham Inspiratory Muscle Training; CT, Cognitive Training; Borg, Borg Dyspnea Scale; COPD, Chronic Obstructive Pulmonary Disease.

**Table 9:** Summary of studies assessing the impact of rehabilitation on quality of life.

Study (Author, Year)	QoL Outcome Measure	Quantitative Results	Narrative Summary
Andrianopoulos et al., 2021 [13]	SF-36	SF-36 Physical Composite Score: CN: 40.0 ± 15.4 → 52.9 ± 17.7 CI: 38.7 ± 20.7 → 48.6 ± 22.8 SF-36 Mental Composite Score: CN: 50.5 ± 17.7 → 60.4 ± 17.1 CI: 46.0 ± 18.2 → 58.5 ± 20.5	After only 3 weeks of PR, both CN and CI patients showed improvements in quality of life. SF-36 Physical and mental scores significantly increased in both groups.
Cheng et al., 2022 [15]	CAT (COPD Assessment Test)—assessed baseline vs. post-intervention.	Total participants pre RMT: CAT 14.17 ± 8.39 (*p* < 0.01). After 8 weeks: CAT 9.06 ± 6.06 (*p* < 0.01). Post-treatment group comparison: Group 1 (IMT + EMT): CAT = 10.00 ± 6.91 Group 2 (IMT): CAT = 7.75 ± 4.46 Difference between groups: *p* = 0.396 (not significant)	Both complete RMT (IMT + EMT) and isolated IMT significantly reduced CAT scores, indicating improved dyspnea and symptom burden. However, no significant difference was observed between training modalities.
France et al., 2021 [16]	CAT and CRQ	AECOPD Group: CAT: ~25 → ~18 CRQ-Dyspnea: ~1.7 → ~2.8 CRQ-Emotion: ~2.8 → ~3.6 CRQ-Mastery: ~2.8 → ~3.6 CRQ-Fatigue: ~2.5 → ~3.3PR PR Group: CAT: ~20 → ~13 CRQ-Dyspnea: ~2.7 → ~4.0 CRQ-Emotion: ~3.6 → ~4.6 CRQ-Mastery: ~3.5 → ~4.5 CRQ-Fatigue: ~3.2 → ~4.0 (All improvements statistically significant, *p* < 0.01)	Both natural recovery (AECOPD) and PR significantly improved dyspnea and quality of life scores. However, PR led to a greater reduction in CAT score (~7 points) and larger increases in all CRQ domains (~+1 to +1.3). This indicates PR is more effective than spontaneous recovery in improving breathlessness and health-related quality of life in COPD.
Kotani et al., 2025 [17]	CAT	CAT: 15.5 ± 6.7 → 15.1 ± 6.2 (after 2 years), *p* = 0.738	Over two years, CAT scores showed no meaningful improvement, despite long-term PR.
Lavoie et al., 2019 [18]	PHQ-9	PHQ-9 (Mean ± SD): Group 1 (T): 4.4 ± 3.6 → 3.1 ± 3.3 Group 2 (T/O): 3.9 ± 3.1 → 2.7 ± 3.0 Group 3 (T/O + Exercise): 4.4 ± 4.0 → 2.7 ± 2.8 Control (SMBM + Placebo): 5.1 ± 4.0 → 3.8 ± 3.9	All treatment groups experienced an improvement in quality of life as reflected by lower PHQ-9 scores after 12 weeks. The greatest improvement occurred in the group receiving combined bronchodilator therapy and exercise training.

Abbreviations: QoL, Quality of Life; SF-36, Short Form-36 Health Survey; PCS, Physical Composite Score; MCS, Mental Composite Score; CN, Cognitively Normal; CI, Cognitive Impairment; CAT, COPD Assessment Test; RMT, Respiratory Muscle Training; IMT, Inspiratory Muscle Training; EMT, Expiratory Muscle Training; PR, Pulmonary Rehabilitation; AECOPD, Acute Exacerbation of Chronic Obstructive Pulmonary Disease; CRQ, Chronic Respiratory Questionnaire; PHQ-9, Patient Health Questionnaire-9; T, Tiotropium; T/O, Tiotropium/Olodaterol; SMBM, Self-Management Behavioural Modification Program; COPD, Chronic Obstructive Pulmonary Disease.

## Data Availability

All data are included in this study.

## Supplementary Materials

The following supporting information can be downloaded at: https://www.mdpi.com/article/10.3390/jcm15020670/s1, File S1. PRISMA_2020_checklist. File S2. PRISMA_2020_abstract_checklist.
